# From Fleming to Endo: The discovery of statins

**DOI:** 10.21542/gcsp.2021.32

**Published:** 2021-12-31

**Authors:** Adrian Chester, Ahmed El Guindy

**Affiliations:** 1 Heart Science Centre, NHLI, Imperial College London, Harefield, Middlesex, UK UB9 6JH; 2 Aswan Heart Centre, Aswan, Egypt

## Abstract

Statins are now one of the most prescribed drugs in the world and have made a major impact to the prevention and treatment of cardiovascular disease, thereby extending the lives of millions of people across the world. The scientist responsible for the discovery of the first statin, Professor Akira Endo, was recently honoured for his ‘exceptional contribution to cardiovascular medicine’ by award of the European Society of Cardiology Gold Medal in 2021. Inspired by the work of Sir Alexander Fleming as a young scientist, Professor Endo pursued a long research career focusing on fungal enzymes and their potential use in medicine; a journey that eventually led him to the discovery of statins.

## The discovery of statins

Frequently dubbed as the most important drug discovery since penicillin, statins revolutionized the prevention and management of atherosclerotic cardiovascular disease and saved millions of lives since their introduction to clinical use in the late 1980s. The class continues to be the amongst the highest selling pharmaceutical agents in history; in fact Lipitor (atorvastatin) remains the best-selling drug of all time with lifetime sales exceeding 150 billion US dollars. This year we celebrate the 50th anniversary of Professor Akira Endo’s pioneering work that eventually led to the discovery of the first statin. Professor Endo was recently honoured for his ‘exceptional contribution to cardiovascular medicine’ by award of the European Society of Cardiology 2021 Gold Medal^1^.

**Figure 1.**
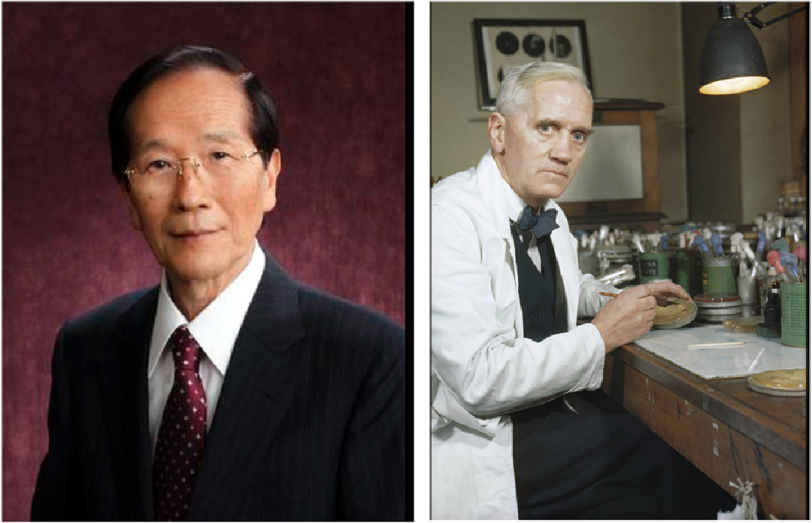
Professor Akira Endo & Sir Alexander Fleming. Akira Endo courtesy of WikiMedia and reproduced under CC BY 4.0 license. Alexander Fleming courtesy of Imperial War Museum and reproduced under their IWM Non Commercial Licence.

From a young age, inspired by his grandfather’s interest science and medicine, Endo became fascinated by mushrooms and mould, and from the age of 10 dreamt of becoming a scientist. While studying at the Faculty of Agriculture at Tokohu University, Endo read Alexander Fleming’s biography and how penicillin was discovered from the mould *Penicillium rubens* (Figure 1). This helped him realise the potential of moulds as a source of pharmacological agents.

After completing a degree, Endo took up a position at the Japanese pharmaceutical company Sankyo in 1957. Initially, worked on the grape-parasitic fungus, *Coniothyrium diplodiella*, isolating a pectinase enzyme that hydrolyzed viscid pectin, which was subsequently commercialised and used in the fermentation process of wines and ciders.

During the 1960’s Endo went to America to study at Albert Einstein College of Medicine in New York City. While living in the Bronx he was struck by two things. Firstly, the number of overweight people and secondly by the number of ambulances he saw attending elderly people who were having heart attacks^2^.

During this period Konrad Bloch and Feodor Lynen were awarded the Nobel Prize for their work on devising the pathway for the synthesis of cholesterol and the epidemiological link was made between cholesterol and coronary artery disease by Ancel Keys from the University of Minnesota^3–5^. In the Seven Countries Study, which followed 15,000 middle-aged men for 10 years, Keys and co-workers were able that show that there was a linear relationship between blood cholesterol levels and the incidence of heart attacks^6–12^. Likewise, the Framingham showed that baseline cholesterol levels were a predictor of subsequent myocardial infarction years later^13^.

**Figure 2.**
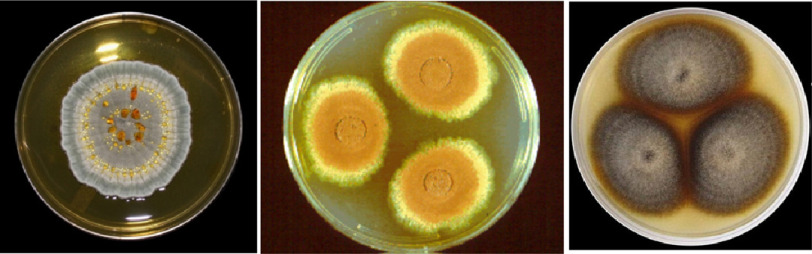
*Penicillium citrinum, Aspergillus terreus* and *Monascus ruber*. (Reproduced with permission by Yuri from *Fun With Microbiology (What’s Buggin’ You?)* http://thunderhouse4-yuri.blogspot.com/2015/08/penicillium-citrinum.html).

Prior to the discovery of statin, there was a limited choice of agents with which to try and lower cholesterol. These ranged from nicotinic acid, which lowered cholesterol and triglycerides, to clofibrate and its derivatives, the fibrates^14,15^. However, these agents produced only a mild-to-moderate reduction in the circulating levels of cholesterol. A more effect strategy was the use of the ion-exchange resin, cholestyramine, which reduced reabsorption of cholesterol from the gut and promoted faecal excretion^16^. While this was highly effective in patient with high levels of cholesterol, it was poorly tolerated in many^17^.

## Patience pays off

In 1968, Endo resumed his position at Sankyo in Japan and choose to look amongst moulds and mushrooms for a source of an enzyme that would inhibit *β*-hydroxy *β*-methylglutaryl-CoA (HMG-CoA) reductase, the enzyme responsible for the rate limiting step in the synthesis of cholesterol (REF).

At that time the method of measuring HMG-CoA reductase activity (incorporation of [^14^C]HMG-CoA into mevalonate) was prohibitively expensive^18^. As an alternative Endo searched for broths of moulds that could convert [^14^C]-acetate into non-saponifiable lipids. Broths that showed activity were then tested for their ability to inhibit lipid synthesis from [^3^H]-mevalonate. Broths that showed activity in the first assay, but not the second were assumed to contain compound(s) with the potential to slow cholesterol synthesis somewhere in the pathway between acetate and mevalonate.

It took a year and the screening of 3,800 fungi before he found a broth with potential activity against inhibition of cholesterol synthesis. The active constituent was found to be a substance called citrinin^19^. Citrinin was subsequently found to be a potent inhibitor of HMG-CoA reductase activity and capable of lowering cholesterol in rats. However, citrinin also show high levels of renal toxicity^2^.

Another year of work passed before a second broth of a blue–green mold, *Penicillium citrinum* was found that showed activity for the inhibition of cholesterol synthesis (Figure 2). The mould in question had been taken from some rice obtained from a local shop in Kyoto, that was similar to the mould commonly found on fruits like oranges. Due to the low productivity of the broth, it took a further year before the active constituents could be isolated. Three compounds were eventually shown to have activity, the most active of these was initially termed ML-236B and later called compactin^20^.

Compactin shared structural similarities with HMG-CoA and proved to be a potent competitive inhibitor of HMG-CoA reductase^20^. Subsequent *in vitro* characterisation of the effect of compactin on culture cells meant it wasn’t until 1976 that Endo was able to publish his findings on the discovery and characterisation of compactin as the first statin^21^. The development of compactin as a drug received a set-back when the compound was tested in rats, where it failed to show any significant effect. It was found that when it was first given there was an initial lowering of serum cholesterol within 8 h, but this effect was lost after repeated dosing^22^. It took a further 2 years to work out that there was a compensatory increase in HMG-CoA reductase expression in the liver of the rats that masked the effect of the drug in the serum. A second set-back was the detection of micro crystals in the livers of rat fed very high doses of compactin, however, these were eventually found to be microcrystalline structures of non-toxic cholesterol^2^.

Towards the end of the 1970s Sankyo started Phase I and II clinical trials with compactin. In the Phase II trial, all participating hospitals reported positive effects of compactin in patients with severe hypercholesterolemia, with an excellent safety profile. However, after the trial was completed, Sankyo halted work on the drug based on the observation that high doses of compactin were associated with development of lymphomas in dogs, even though these very high doses were unlikely ever to be used clinically^2^.

Inspired by Endo’s work at Sankyo, several other pharmaceutical companies started to search for compounds with cholesterol lowering properties. Meanwhile Endo relocated to the Tokyo University of Agriculture and Technology where he continued his own work. Independently, researchers from Merck and Endo isolated a compound with HMG-CoA reductase inhibitory activity form *Aspergillus terreus* and *Monascus ruber* respectively^22,23^. While, these compounds were initially given different names, further work showed them to have the same chemical structure, which was then named lovastatin.

Progress with lovastatin was initially stalled due to the news of the development of cancer in dogs with compactin due to the similarities in chemical structure with lovastatin. However, observations that showed lovastatin induced an up regulation of LDL receptors and a large fall in plasma LDL, an effect that was also shown to be shared with compactin^24^. These reports rekindled interest in lovastatin and in 1982 researchers from the University of Texas started studies in humans with hypercholesteremia, which showed dramatic activity in lowering LDL cholesterol^25^. Soon after Merck started large scale clinical trials with lovastatin which confirmed the effects on reducing cholesterol levels and showed the drug to be well tolerated with no evidence of tumors^26^. In September 1987 lovastatin was approved by the FDA and became the first statin available for clinical use.

**Figure 3.**
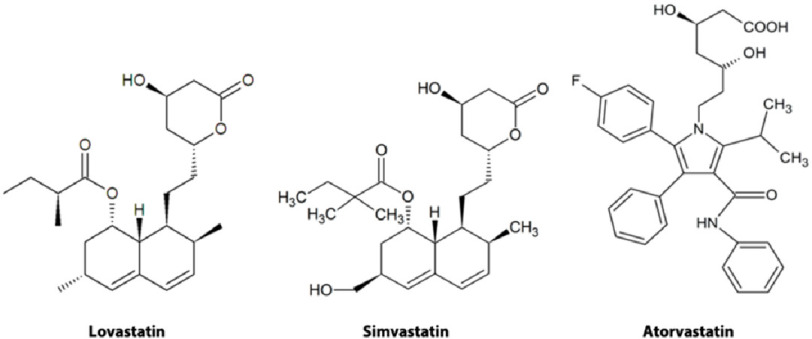
Chemical structure of natural, semi-synthetic, and synthetic statins. Reproduced under CC BY licence from *J. Fungi* **2016**, *2*(2), 13; https://doi.org/10.3390/jof2020013.

Since the approval for the use of lovastatin, several naturally occurring and some synthetic statins have been developed and are in clinical use^27^ (Figure 3). The vision and determination shown by Professor Endo to develop this class of drug serves as an excellent example of the commitment and perseverance required for the discovery of new drugs. His work laid the foundations for drugs that have made a significant impact in our ability to prolong the lives of patients with cardiovascular disease.
